# Breaking of Plant Stomatal One-Cell-Spacing Rule by Sugar Solution Immersion

**DOI:** 10.1371/journal.pone.0072456

**Published:** 2013-09-11

**Authors:** Kae Akita, Seiichiro Hasezawa, Takumi Higaki

**Affiliations:** 1 Department of Integrated Biosciences, Graduate School of Frontier Sciences, The University of Tokyo, Kashiwanoha, Kashiwa, Chiba, Japan; 2 Advanced Measurement and Analysis, Japan Science and Technology Agency, Chiyoda-ku, Tokyo, Japan; University College Dublin, Ireland

## Abstract

The spatial distribution of plant stomata is a model system to study epidermal cell pattern formation. Molecular genetic approaches have identified several key genes required for stomatal distribution patterning, but environmental conditions that perturb the stomatal spacing distribution have not yet been identified. We found that immersing hydroponic cultures in 1–5% sucrose solution induced abnormally clustered stomata in the cotyledons of Arabidopsis seedlings. Clustered stomata were also induced by treatment with glucose or fructose solution but not by mannitol solution, suggesting that osmotic stress was not a cause of the disturbed stomatal patterns. Stomatal lineage cell-specific enhancer trap lines revealed that the sugar solution treatment led to ectopic expression of stomatal lineage cell-specific genes in non-stomatal lineage cells. Aniline blue staining also showed that there was reduced deposition of callose, a plant cell wall component, in new cell walls during formation of stomatal precursor cells (meristemoids). These results suggested that the immersion treatment with sugar solution permitted ectopic guard cell differentiation through dysfunction of the cell wall dividing stomatal- and non-stomatal lineage cells. Our simple induction system for clustered stomata provides a suitable tool for further studies to investigate the one-cell-spacing rule during plant stomatal development.

## Introduction

Plant stomata are pores enabling gas exchange and transpiration on the aerial plant body surface that are formed by a pair of kidney-shaped epidermal cells named guard cells. There are very few guard cell pairs in direct contact with one another, and the spatial regularity of these differentiated cells is known as the ‘one-cell-spacing rule’ [1]. This one-cell-spacing rule is thought to help ion and/or water exchange between guard cells and neighboring epidermal cells during stomatal movement. Screening of Arabidopsis stomatal distribution mutants has identified several genes that positively or negatively regulate guard cell differentiation. Gene identification and functional analysis has suggested that guard cell differentiation is negatively regulated by EPIDERMAL PATTERNING FACTORs (EPFs) [2], [3], which are putative ligand peptides secreted from stomatal lineage cells, in cooperation with the putative receptor TOO MANY MOUTHS (TMM) [4], [5] or ERECTA-family leucine-rich-repeat receptor-like kinases [6]. Downstream MAPK cascades including YODA (MAPKKK) [7], MKK4/5 (MAPKK) [8] and MPK3/6 (MAPK) [8] inactivate the heterodimeric transcription factor SPEECHLESS (SPCH)/ SCREAM (SCRM) [9], [10] or MUTE/SCRM [10], [11] that positively regulates guard cell differentiation. A positive regulator of guard cell differentiation STOMAGEN/EPFL9, a secreted peptide from mesophyll cells, has been identified [12], [13]. It is also suggested that there is an intrinsic mechanism to ensure appropriate spacing. BREAKING OF ASYMMETRY IN THE STOMATAL LINEAGE (BASL) is an essential regulator of the unequal cell division that separates stomatal lineage cells and non-stomatal lineage cells with cell periphery localization distal to the unequal division plane, that is, dysfunction of BASL results in direct contact between stomatal precursor cells, meristemoids, and the subsequent guard cells [14]. The localization and functions of BASL in unequal cell division plane determination is independent of putative ligands for stomatal-derived signaling, TMM or EPF1 [14]. In addition, cell wall integrity is also suggested to be important for the one-cell-spacing rule. GLUCAN SYNTHASE-LIKE 8 (GSL8)/CHORUS (CHOR) is a putative synthase of callose, which is a homopolysaccharide that is abundant in the cell plate and new cell wall during plant cytokinesis [15], [16]. Dysfunction of GSL8/CHOR leads to stomatal clusters in direct contact, implicating the leakage of cell fate determinants or other regulatory factors [16].

As described above, mutant studies have given us the molecular basis for plant stomatal spacing patterns. However, as far as we know, there have been no reports about conditions or treatments of non-gene products that disturb the stomatal one-cell-spacing rule. We believe that establishment of an experimental system to perturb the one-cell-spacing rule would also promote our knowledge of plant stomatal pattern formation. Here, we report that sugar solution immersion induces clustered stomata in Arabidopsis seedlings, and discuss its implications.

## Materials and Methods

### Plant growth conditions

Sterilized Arabidopsis wild-type seeds (Col-0) or enhancer trap lines E1728 and E1627 [17] were grown in 1/2 Murashige-Skoog media solution [2.3 mg L^−1^ Murashige and Skoog Plant Salt Mixture (Code No. 392–00591: Wako Pure Chemical Industries, Osaka, Japan; http://www.wako-chem.com/)] (pH 5.8) supplemented with or without sucrose (Code No. 193–00025: Wako Pure Chemical Industries), glucose (Code No. 049–31165: Wako Pure Chemical Industries), fructose (Code No. 127–02765: Wako Pure Chemical Industries) or mannitol (Code No. 130–00855: Wako Pure Chemical Industries) using a 24-well plate (Sumilon Multi Well Plate, Sumitomo Bakelite; http://www.sumibe.co.jp/) in a growth chamber at 23.5°C with a 12 h light/12 h dark cycle (100 μmol m^−2^ s^−1^ white light).

### Cell staining

To visualize plasma membranes, leaves were immersed in basal buffer [5 mM MES-Tris, 10 mM CaCl_2_, 50 mM KCl, pH 6.5] supplemented with 32 μM FM4-64 (Life Technologies; http://www.lifetechnologies.com) for 10 min. For callose staining, 4 day-old seedlings were immersed in basal buffer supplemented with 0.02% (w/v) aniline blue for 1 week at 4°C [18].

### Microscopy and image analysis

To acquire confocal images, we used a fluorescence microscope (IX-71; Olympus; http://www.olympus.com) equipped with a confocal laser scanning head and control system (FLUOVIEW FV300; Olympus), together with a sapphire laser (488 nm; Showa Optronics; http://www.soc-ltd.co.jp) and a helium-neon laser (543 nm; Showa Optronics). Maximum intensity projection images were reconstructed from serial optical sections obtained at 0.5 μm intervals with the ImageJ software. The clustered stomata were manually counted using the ImageJ interface. To detect the fluorescence from aniline blue, we used a fluorescence microscope (BX; Olympus) equipped with a CCD camera system (DP70; Olympus).

## Results and Discussion

**Figure 1:**
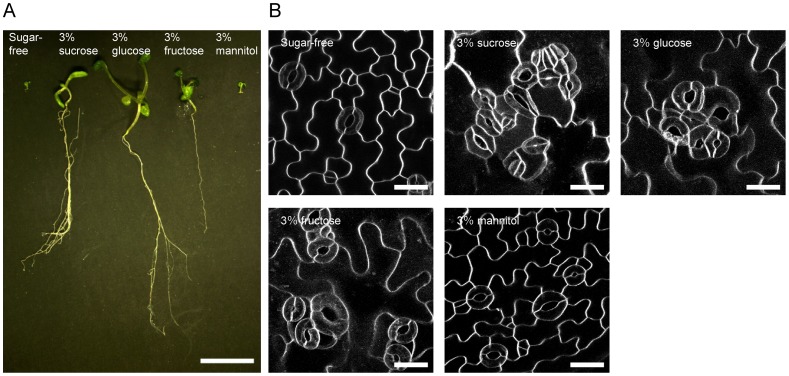
Effects of immersion in sugar-free, 3% sucrose, glucose, fructose and mannitol solutions on Arabidopsis seedlings and cotyledon guard cell distributions. (A) Representative 14 day-old seedlings. Scale bar  = 1 cm. (B) Fluorescence microscopy images of abaxial cotyledon epidermis stained with FM4-64 dye. Representative images from 10–24 independent seedlings were shown. The maximum intensity projections were constructed with a 0.5 µm step size. Scale bars  = 20 μm.

**Figure 2:**
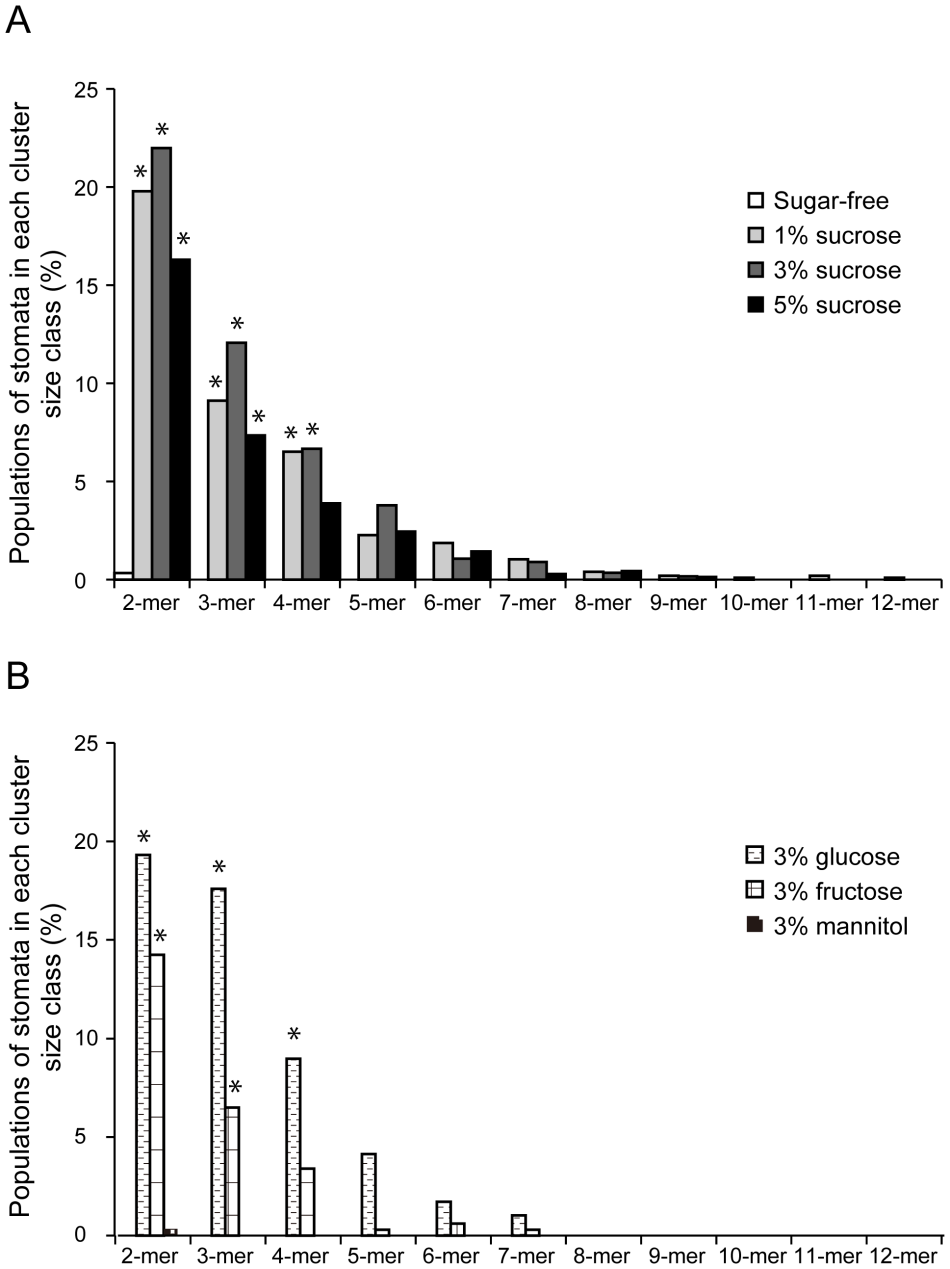
Percentage of stomata in each cluster size class. Abaxial cotyledons from 12- to 15 day-old seedlings grown in sugar-free, 1, 3 or 5% sucrose (A), and 3% glucose, fructose or mannitol (B) solutions were subjected to quantitative analysis. Data are mean values of 20–68 independent observations. Significance with sugar-free conditions was determined using Mann–Whitney's U-test. *p*-value *<0.0001. Total number of stomata counted: *n* = 281–1843.

Sterilized Arabidopsis seeds were sowed in a 24-well plate filled with sugar-free, 3% sucrose, glucose, fructose and mannitol solutions to immerse the emerged cotyledons. The sugar solution treatment enhanced seedling growth and thickened the cotyledons of 12- to 15 day-old seedlings (Figure 1A). Visualization of plasma membranes of the cotyledon epidermis with the fluorescent dye FM4-64 showed that clustered stomata were frequent in the seedlings immersed in 3% sucrose, glucose or fructose (Fig. 1B). In the cotyledon epidermis of seedlings immersed in 1–3% sucrose solution, stomatal pairs (16.3–22.0%), trios (7.36–12.1%), quartets (6.67–3.90%) and clusters of 5–12 adjacent stomata (6.31–4.76%) were observed (Figure 2A). Comparable stomata frequencies were confirmed in the glucose and fructose treatments (Figure 2B), and there were highly significant differences with sugar-free conditions (*p*-value <0.0001, Mann–Whitney's U-test). The stomatal density also significantly increased about 2–3 times after sucrose, fructose or glucose solution immersion compared with sugar-free conditions (Figure S1). The clustered stomata were also induced in true leaves immersed in 3% sucrose solution (Figure S2A). Clustered stomata on cotyledons were rarely observed in sugar-free control conditions (Figures 1B and 2A, sugar-free) or 3% mannitol solution (Figures 1B and 2B, 3% mannitol). These results suggested that clustered stomata formation was not a result of osmotic effects. In the cotyledon epidermis of seedlings grown on a gellan gum plate supplemented with 3% sucrose, the stomata were very rarely clustered (Figure S2B). This observation suggested that direct exposure of the cotyledon epidermis to the solution was required for sugar-induced breaking of the stomatal one-cell-spacing rule.

**Figure 3:**
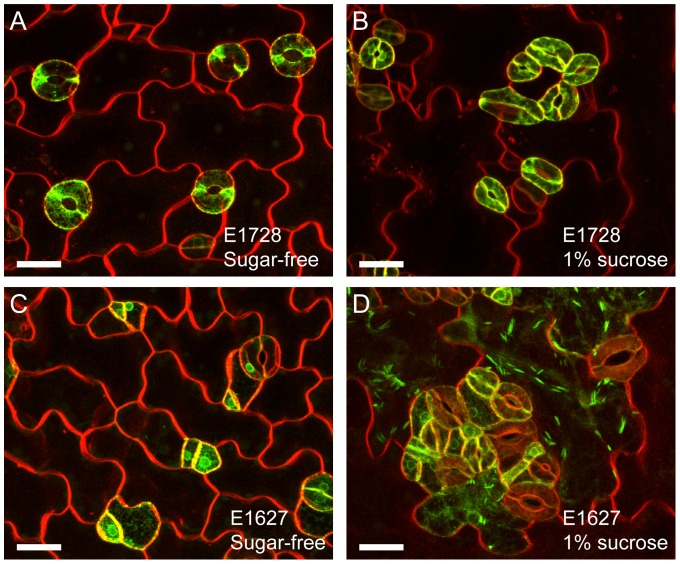
Effects of sucrose exposure on stomatal lineage cell markers. (A and B) Mature guard cell marker E1728-labeled [15] and FM4-64-stained cotyledon epidermis from sugar-free control (A) and 1% sucrose (B) treatments. (C and D) Stomatal cell lineage marker E1627-labeled [15] and FM4-64-stained cotyledon epidermis from sugar-free control (C) and 3% sucrose (D) treatments. Representative images from 10–15 independent seedlings were shown. Note that the jigsaw puzzle-shaped epidermal cells were labeled with E1627 in the sucrose treatment but not in the sugar-free control. Scale bars  = 20 μm.

To check whether the clustered kidney-shaped cells genetically progressed into mature guard cells, we next observed the cotyledons of the GAL4 GFP enhancer trap line E1728, in which mature guard cells are specifically labeled by GFP with an endoplasmic reticulum (ER)-retention signal [17], [19]. In the sucrose treatment, the clustered cells showed variations in size but GFP signals were clearly detected (Figure 3B); GFP signals were also detected in the no sugar treatment (Figure 3A). The enhancer trap line E1627 labels stomatal lineage cells including meristemoids, guard mother cells and guard cells [17] (Figure 3C). Interestingly, E1627 labeled the jigsaw puzzle-shaped epidermal cells in sucrose-treated cotyledon epidermis (Figure 3D), with a cigar-shaped ER-body (an ER-derived structure) [20]. In the control sugar-free solutions, the jigsaw puzzle-shaped epidermal cells were never labeled with GFP-ER in the E1627 line (Fig. 3C). These results suggested that sucrose treatment conferred the jigsaw puzzle-shaped epidermal cells with stomatal lineage-like gene expression patterns. This feature suggests a working hypothesis that sugar solution immersion induces leakage of unidentified guard cell-fate determinants into non-stomatal lineage cells, much like in a previous report on *gsl8/chor* mutants that showed similar clustered stomata phenotypes [16]. GSL8/CHOR was suggested to positively regulate callose synthesis at the expanding cell plate, because reduced callose deposition was observed at the new cell wall in the *gsl8/chor* mutant [15], [16]. New cell walls with reduced callose have been suggested to be unable to contain cell-fate determinants in stomatal lineage cells [16].

**Figure 4:**
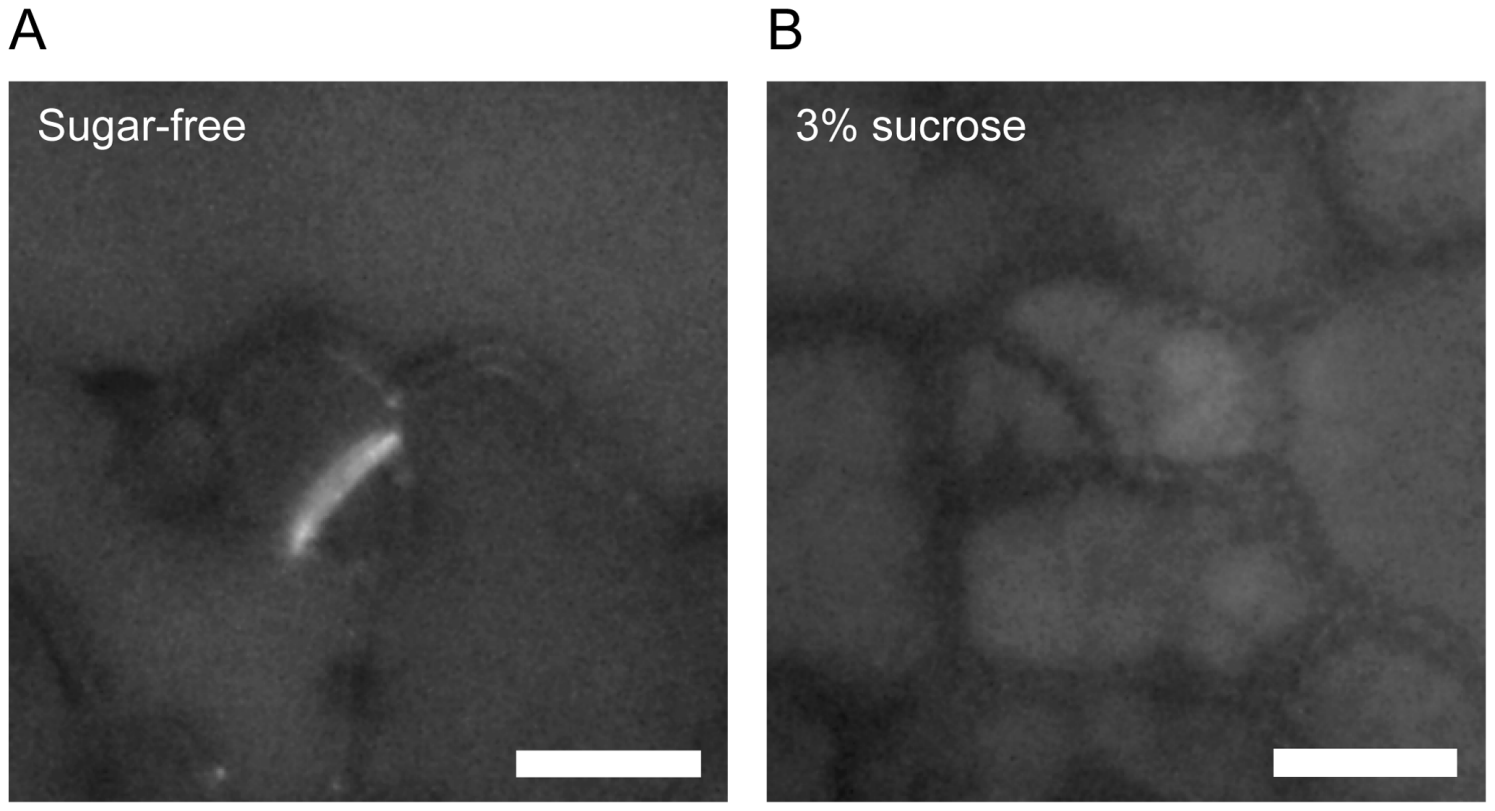
Aniline blue staining of cotyledon epidermal cells. Four day-old cotyledons immersed in sugar-free (A) or 3% sucrose (B) solutions were stained with 0.02% aniline blue for 1 week. Representative images from 24 (sugar-free) and 38 (3% sucrose) independent seedlings were shown. Note that aniline blue fluorescence was clearly detected in new cell walls forming in meristemoids immersed in sucrose-free solutions but not in 3% sucrose solutions. Scale bars  = 10 μm.

Therefore, we next investigated the effects of sugar solution immersion on callose deposition in newly synthesized cell walls in the cotyledon epidermis by aniline blue staining [18]. To efficiently observe new cell walls, younger 4 day-old seedlings were used (Figure S3). Aniline blue fluorescence was clearly detected from new cell walls separating meristemoids and epidermal cells in the control (Figure 4A). However, we could not detect significant fluorescence from aniline blue in new cell walls of samples treated with sucrose solution (Figure 4B). These results suggested that immersion in sugar solution suppresses callose deposition during unequal cell division in the cotyledon epidermis as previously reported in the *gsl8/chor* mutant [15], [16]. One possible reason why sugar treatment results in reduced callose deposition is substrate inhibition of sucrose synthase (SuSy). It has been suggested that a kind of SuSy is coexpressed with callose synthase and catalyzes the reversible conversion of sucrose and UDP to UDP-glucose and fructose, supplying UDP-glucose for synthesis of cell wall components including callose [21], [22]. SuSy is reported to be inhibited in the UDP-glucose synthetic direction by high concentrations of the substrate fructose or UDP-glucose [23], [24]. In our experimental system, immersion in sugar solutions might possibly have induced substrate inhibition of UDP-glucose producing SuSy activity *in vivo*, but further studies are necessary to clarify the relationship between carbohydrate metabolism and the establishment of the stomatal one-cell-spacing rule.

## Conclusions

We have established an experimental system for reproducible induction of clustered stomata in Arabidopsis seedlings by immersion into sucrose, glucose or fructose solution. Sugar solution immersion reduced callose deposition during the unequal cell division that separates stomatal lineage cells and nonstomatal lineage cells, and the compromised cell wall integrity might result in guard cell-fate determinant leakage and clustered stomata. This easy hydroponic culture system using sucrose solution may help to identify and/or monitor the determinants or regulatory proteins that establish stomatal or nonstomatal cell fate in future studies.

## Supporting Information

Figure S1 Effects of immersion in sugar-free, 1, 3 or 5% sucrose, 3% glucose, fructose and mannitol solutions on stomatal density. Data are mean values ± SD of 20–59 independent observations. Significance with sugar-free conditions was determined using Mann–Whitney's U-test. *p*-value *<0.0001. Total number of stomata counted: *n* = 281–1843.
(TIF)

Figure S2 **Effects of sugar treatment on stomatal distributions.** (A) Stomatal distribution in the true leaf epidermis of seedlings grown immersed in 3% sucrose solution. Representative images from 10 independent seedlings were shown. (B) Stomatal distribution in the cotyledon epidermis of seedlings grown on a gellan gum plate supplemented with sugar-free (left) or 3% sucrose (right) solution. Representative images from 20 independent seedlings were shown. Scale bars  = 20 μm.
(TIF)

Figure S3 **Representative seedlings for aniline blue observations.** Four day-old seedlings with sugar-free (left) or 3% sucrose (right) solution were stained with 0.02% aniline blue for 1 week and then observed. Representative images from 24 (sugar-free) and 38 (3% sucrose) independent seedlings were shown. Scale bar  = 1 mm.
(TIF)
