# Supplementary figures and images for: Breaking of Plant Stomatal One-Cell-Spacing Rule by Sugar Solution Immersion
Source: PLoS One. 2013 Sep 11;8(9):e72456. doi: 10.1371/journal.pone.0072456 (PMC3770691; doi:10.1371/journal.pone.0072456)

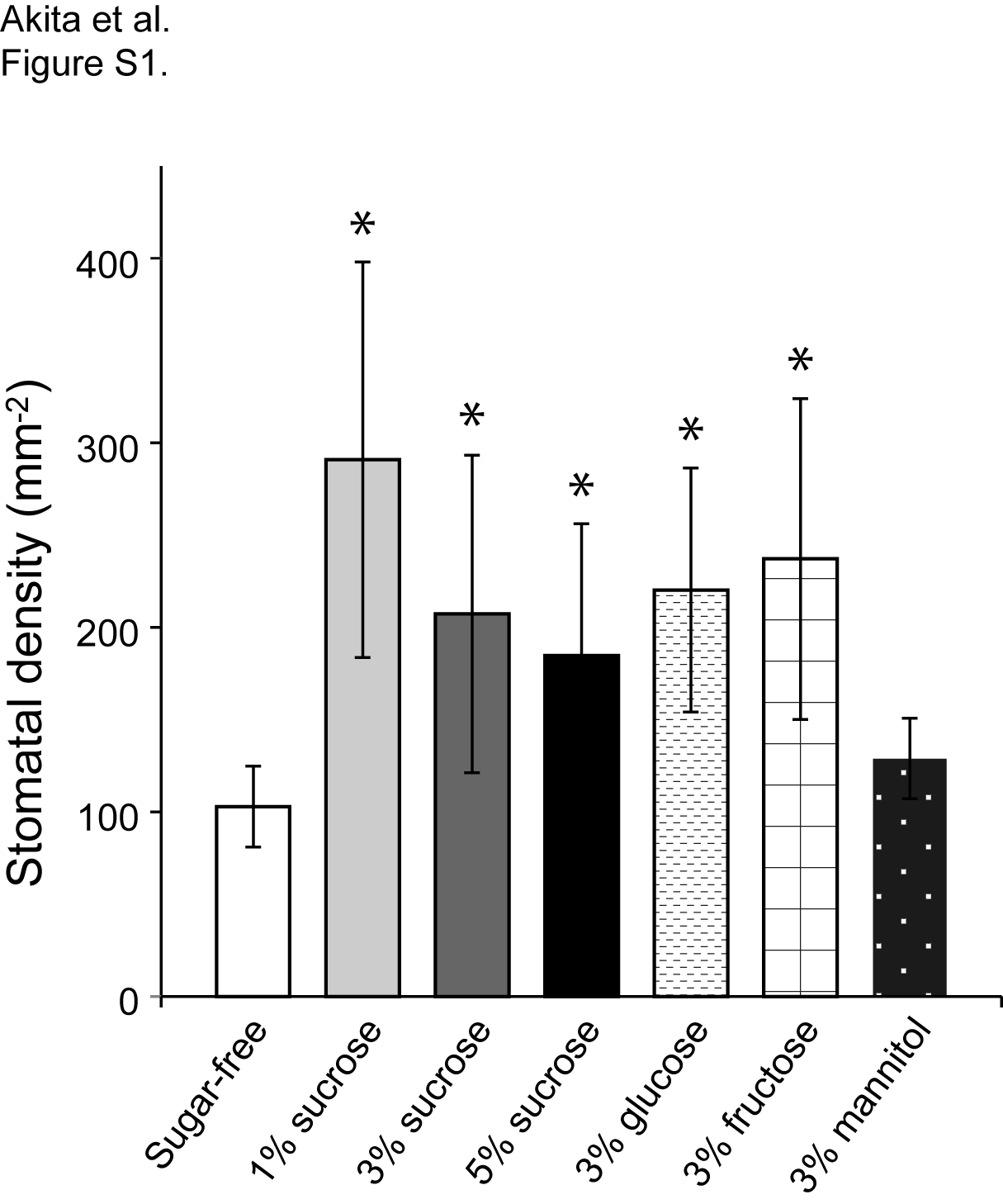

Supplement: Figure S1 — Effects of immersion in sugar-free, 1, 3 or 5% sucrose, 3% glucose, fructose and mannitol solutions on stomatal density. Data are mean values ± SD of 20–59 independent observations. Significance with sugar-free conditions was determined using Mann–Whitney's U-test. p-value *<0.0001. Total number of stomata counted: n = 281–1843. (TIF) [file pone.0072456.s001.tif]

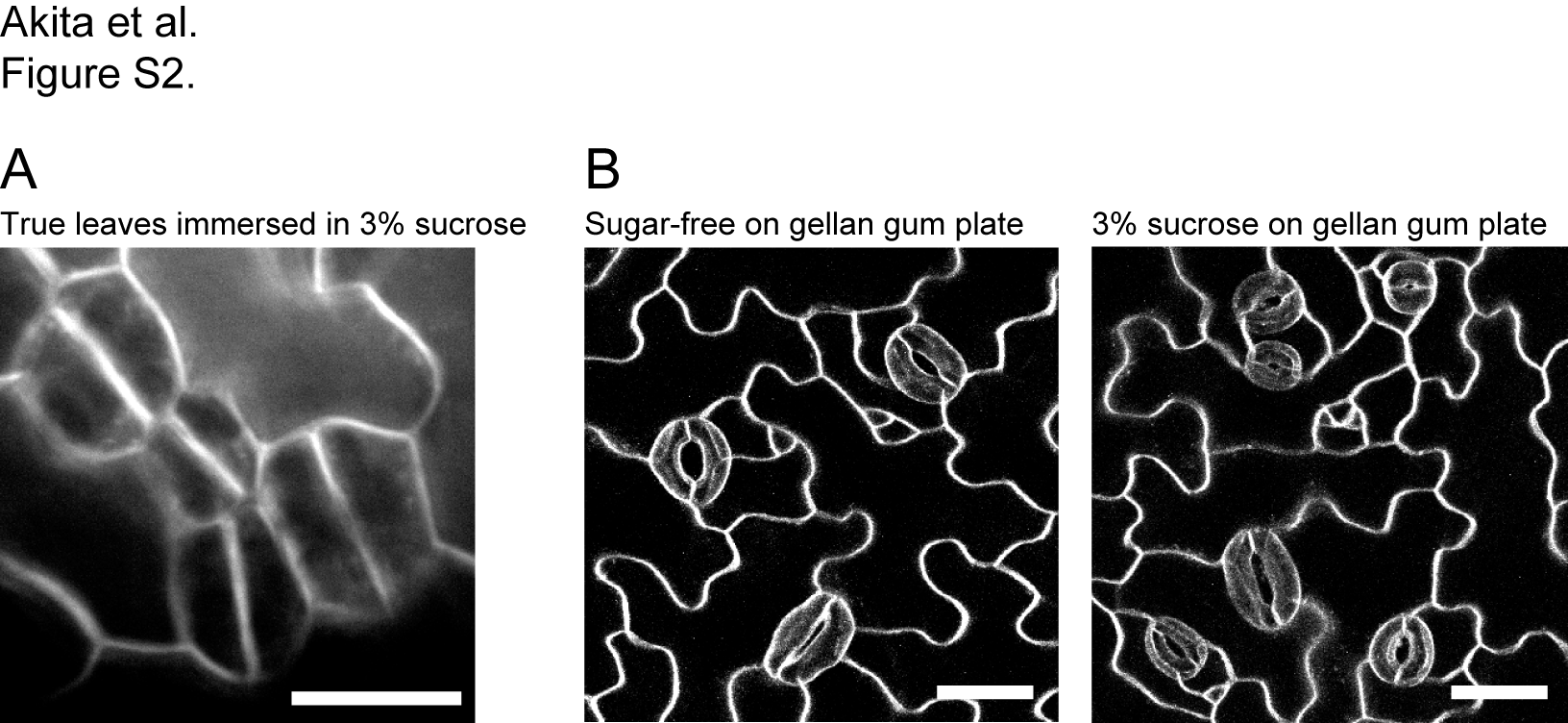

Supplement: Figure S2 — Effects of sugar treatment on stomatal distributions. (A) Stomatal distribution in the true leaf epidermis of seedlings grown immersed in 3% sucrose solution. Representative images from 10 independent seedlings were shown. (B) Stomatal distribution in the cotyledon epidermis of seedlings grown on a gellan gum plate supplemented with sugar-free (left) or 3% sucrose (right) solution. Representative images from 20 independent seedlings were shown. Scale bars = 20 μm. (TIF) [file pone.0072456.s002.tif]

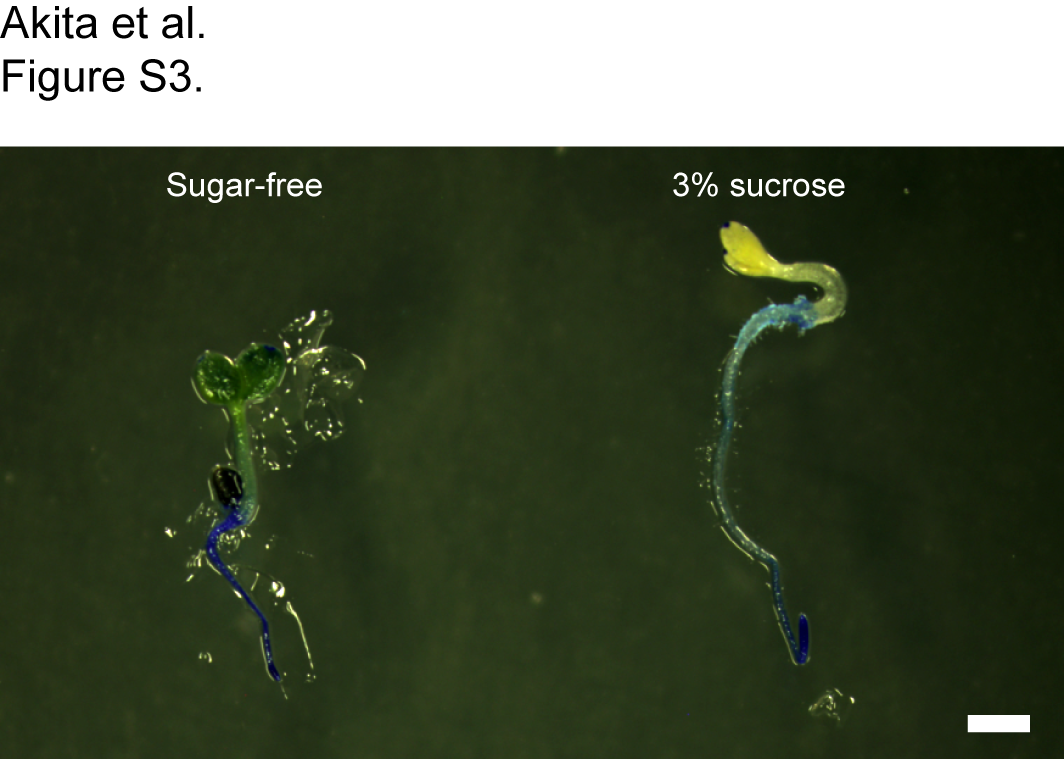

Supplement: Figure S3 — Representative seedlings for aniline blue observations. Four day-old seedlings with sugar-free (left) or 3% sucrose (right) solution were stained with 0.02% aniline blue for 1 week and then observed. Representative images from 24 (sugar-free) and 38 (3% sucrose) independent seedlings were shown. Scale bar = 1 mm. (TIF) [file pone.0072456.s003.tif]
